# Adult-onset congenital intestinal malrotation: A case report and literature review

**DOI:** 10.1097/MD.0000000000037249

**Published:** 2024-02-23

**Authors:** Meng-Di Yin, Li-Liang Hao, Guang Li, Yu-Tao Li, Bao-Li Xu, Xin-Rui Chen

**Affiliations:** a Linyi People’s Hospital, Jinzhou Medical University, Linyi 276000, China; b Department of Gastrointestinal Surgery, Linyi People’s Hospital, Linyi 276000, China

**Keywords:** adult, bowel obstruction, case report, congenital intestinal malrotation, intestinal volvulus, Ladd Procedure

## Abstract

**Background:**

Intestinal malrotation is an infrequent congenital anomaly primarily observed in neonates, and adult-onset cases are exceedingly rare. Studies on adult congenital intestinal malrotation are limited.

**Methods:**

A case with congenital intestinal malrotation is reported in our study. The clinical data were collected and the treatment process and effect were evaluated.

**Results:**

A 45-year-old female who had been experiencing vomiting for over 40 years was admitted to our hospital. According to the result of CT scan, intestinal volvulus accompanied by bowel obstruction was suspected. Then laparoscopic examination was applied to the patient and was ultimately diagnosed with adult congenital intestinal malrotation. We performed Ladd’s procedure combined with gastrojejunostomy and Braun anastomosis. The patient recovered well and was successfully discharged from the hospital on the 13th day after surgery. After a 6-month follow-up, the symptom of vomiting was significantly alleviated and body weight was gained for 10 kg. She was very satisfied with the treatment.

**Conclusion:**

Adult congenital intestinal malrotation is a rare disease that is often misdiagnosed owing to nonspecific clinical manifestations. Therefore, awareness about this condition should be enhanced. Surgery remains the cornerstone of treatment for this disease. Combining gastrojejunostomy and Braun anastomosis with the traditional Ladd procedure can optimize surgical outcomes.

## 1. Introduction

Intestinal malrotation refers to a congenital anomaly in which embryonic development hinders the rotation of the intestine, specifically around the superior mesenteric artery. This hindrance results in the abnormal positioning of the intestines and the incomplete attachment of the mesentery, leading to duodenal obstruction and small bowel volvulus. The inadequate attachment of the mesentery forms the pathological basis for midgut volvulus.^[1,2]^ Intestinal malrotation is uncommon in adults and comprises merely 0.2% to 0.5% of the cases reported in the literature.^[3–7]^ Therefore, a retrospective analysis was conducted on one case of adult congenital intestinal malrotation treated at our hospital. Moreover, pertinent literature was reviewed to enhance awareness, facilitate timely and accurate diagnosis and treatment, and ultimately improve the prognosis of patients with this condition.

### 1.1. Case presentation

#### 1.1.1. Chief complaints

A 45-year-old woman presented to our hospital with a complaint of vomiting for over 40 years, which had worsened in the past week.

#### 1.1.2. History of the present illness

The patient reported experiencing nausea and vomiting approximately 3 hours after consuming sweets or acidic foods since childhood. The vomitus appeared bilious in nature. During the past week, she did not experience any accompanying symptoms, such as diarrhea, abdominal pain, pallor, or diaphoresis. Bowel movements and passing gas were normal. Previous examination at an external clinic suggested intestinal obstruction, for which intravenous fluids and enemas were administered. However, these treatments provided only temporary relief, and the episodes recurred. She visited our hospital seeking further diagnosis and treatment options. She was admitted as an inpatient with a provisional diagnosis of “intestinal obstruction.” Since the onset of this illness, the patient had normal appetite and sleep patterns. Nonetheless, she experienced a drastic weight loss of approximately 10 kg over the past week.

#### 1.1.3. History of past illness

The patient’s medical history included a diagnosis of gastroesophageal reflux disease and duodenal ulcer, which were identified during an esophagogastroduodenoscopy performed before the month of October. Additionally, colitis was diagnosed via a colonoscopy performed before August. Furthermore, the patient had undergone thyroidectomy previously and reported an allergy to cephalosporin antibiotics.

#### 1.1.4. Personal and family history

The patient denied any family history of genetic disorders.

#### 1.1.5. Physical examination upon admission

The abdominal wall appeared flat with no visible varicose veins. A distinct intestinal pattern was observed on the right side of the abdomen. Palpation revealed a soft abdomen, with tenderness present in the lower right quadrant without rebound tenderness or muscle guarding. Liver, spleen, or gallbladder were not palpable below the ribs. Murphy sign was not present, and tenderness was not observed upon percussion over the liver, spleen, and kidney areas. Absence of shifting dullness was noted. Bowel sounds were auscultated at the rate of 6 per minute, with slightly increased intensity. Edema was not detected in either lower extremities.

#### 1.1.6. Laboratory examinations

Complete blood count yielded the following results: white blood cell count 12.81 × 10^9^/L, with neutrophils accounting for 77.5%; red blood cell count 5.16 × 10^12^/L; hemoglobin level 157 g/L; and platelet count 274 × 10^9^/L. Significant abnormalities were not observed in coagulation parameters, blood biochemistry, or tumor markers.

#### 1.1.7. Imaging examinations

Plain abdominal computed tomography (CT) scan revealed whirlpool-like alterations in a localized section of the small intestine, accompanied by significant dilation in the proximal segment of the intestinal tube. These findings suggested the potential occurrence of small bowel volvulus and subsequent intestinal obstruction.

**Figure 1.**
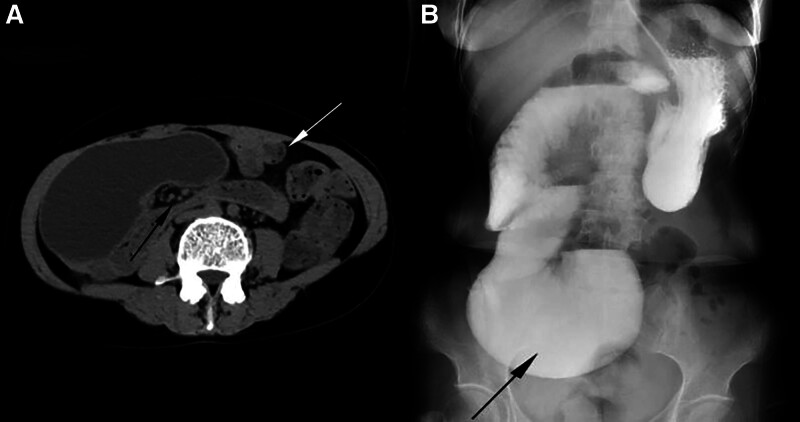
Imaging manifestation. (A) The appendix is situated in the upper left quadrant of the abdomen (indicated by the white arrow) and exhibits a torsion, presenting as a “vortex-like alteration”; (B) Small bowel barium examination reveals an enlarged duodenum during a digestive tract contrast evaluation.

Angiography of the entire digestive tract revealed significant intestinal dilatation, which was indicative of small intestinal obstruction (Fig. 1).

### 1.2. Additional diagnostic work-up

After admission, the patient underwent various symptomatic treatments, which included gastric and intestinal decompression, acid suppression, and total parenteral nutrition. However, her symptoms were not significantly alleviated. Considering the possibility of strangulated intestinal obstruction and subsequent lethal bowel necrosis as well as inadequate response to conservative treatment, laparoscopic examination was performed. During surgery, severe adhesions were evident in the ileum, ascending colon, and appendix located in the lower left abdomen. As laparoscopic detachment could be challenging, a decision was made to transition to an open surgical approach.

Further investigations revealed that the small intestine had undergone clockwise torsion along the mesenteric artery, which resulted in substantial membrane adhesions and proximal compression of the duodenum. Consequently, a duodenal obstruction occurred, which was characterized by obvious dilation of the intestinal tube by up to 15 cm at its widest point, protruding toward the lower right abdomen.

### 1.3. Final diagnosis

Based on the patient’s medical history, physical examination, imaging studies, and surgical findings, the final diagnosis was confirmed as “intestinal malrotation.”

### 1.4. Treatment

**Figure 2.**
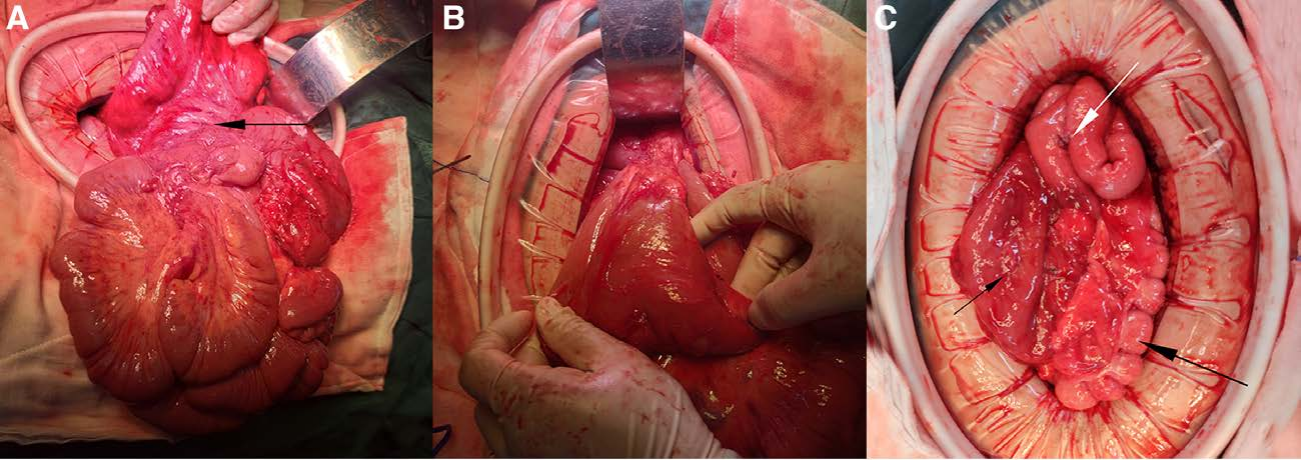
Intraoperative observations and findings. (A) The Ladd band, situated between the cecum and duodenum, exerts pressure on the duodenum, resulting in the clockwise rotation of the small intestine. In addition, the cecum, ascending colon, and appendix are positioned within the left abdomen; (B) After the release of adhesions, a noticeable dilation and descent of the duodenum were recorded; (C) The post-anastomosis findings included an expanded duodenum (indicated by a short arrow), the placement of the cecum left abdominal cavity (indicated by a long arrow), and the removal of the appendix and Braun anastomosis (highlighted by a white arrow).

Ladd procedure was performed to release the adhesion band, and the twisted small intestine was repositioned in an organized manner within the right abdominal cavity. Simultaneously, the cecum and ascending colon were relocated to the left abdominal cavity, and the dilated distal small intestine was subjected to gastrojejunostomy with the greater curvature of the stomach at a point 10 cm below the anastomosis. Additionally, side-to-side anastomosis (Braun anastomosis) was performed between input and output intestinal loops. As a preventive measure for appendicitis diagnosis and treatment, appendectomy was also performed during the surgery (Fig. 2).

### 1.5. Outcome and follow-up

The gastric tube was removed on postoperative Day 5, followed by intra-abdominal drainage removal on Day 7. The patient recovered smoothly and was discharged on Day 13 without any gastrointestinal symptoms, such as nausea or vomiting. Follow-up telephonic interviews were conducted at 3 and 6 months, which revealed the absence of digestive complications. The patient also demonstrated an approximate weight gain of 10 kg during this period.

## 2. Discussion

Although congenital intestinal malrotation is frequently observed in newborns, its occurrence in adults is extremely rare, with reported prevalence rates ranging from 0.2% to 0.5% only.^[3,5–7]^ Normal embryonic development involves a complex process wherein the intestines assume a straight-tube shape by the 4th week of gestation. By the 5th week, the formation of blood vessels is initiated, which divides the intestines into 3 distinct parts: foregut, midgut, and hindgut. Intestinal rotation primarily affects the midgut and progresses via 3 stages. During the first stage, that is, from the 5th to 10th week, the midgut protrudes outward from the embryo’s body cavity, followed by a counterclockwise rotation of 90° around the superior mesenteric artery before returning to the abdominal cavity. During the second stage, which occurs approximately in the 11th week, further counterclockwise rotation of 180° occurs. This process completes the total counterclockwise rotation of 270° over the 2 stages. This rotation results in the formation of a C ring behind the superior mesenteric artery, with the ascending colon positioned on the right side, transverse colon above it, and the descending colon on the left side. The third stage involves fusion and fixation between the midgut mesentery and posterior abdominal wall. This stage also encompasses the descent of the cecum to the right side along with the attachment of the small intestine, ascending colon, and descending colon to the posterior abdominal wall.^[8–10]^ An obstruction occurring at any of these stages can result in gastrointestinal abnormalities. For instance, first-stage abnormality is caused by the inability of the intestinal tube to return to the abdominal cavity, which results in umbilical hernia. Abnormalities in the second stage include intestinal malrotation, abnormal rotation, and reverse rotation. Third-stage abnormalities involve unattached duodenum, small bowel mesentery, and movable appendix.^[8,9]^ Rotational obstruction during the second and third stages may lead to an abnormal Ladd band or the compression of the appendix and ascending colon on the duodenum, resulting in intestinal obstruction. This issue could also cause acute small bowel obstruction owing to torsion of the hypermobile small bowel mesentery. In severe cases, vascular disorders may result in ischemic necrosis of the small intestine.^[10]^

In infants, bilious vomiting, abdominal distension, bloody stools, and delayed development are the most prevalent symptoms of malrotation.^[11–14]^ On the contrary, in adult patients, clinical manifestations tend to be diverse and are often nonspecific, encompassing acute, chronic acute, chronic, and incidental symptoms.^[3,15–17]^ Adult congenital intestinal malrotation typically presents as intestinal obstruction, which is accompanied by malnutrition and intestinal necrosis in some cases.^[18]^ A study has observed that only approximately 20% of the patients exhibit acute symptoms.^[15,19]^ The principal manifestations are recurrent gastrointestinal problems,^[20]^ which are often characterized by intermittent severe abdominal pain, intractable vomiting, hematemesis (vomiting blood), and constipation. Some patients may experience malnutrition and weight loss, and others may develop peritonitis because of intestinal strangulation and hemorrhagic necrosis.^[18]^ Additionally, certain patients seek medical aid owing to acute bowel obstruction with or without a history of previous abdominal surgery.^[20]^ These symptoms could be attributed to the compression of the small intestine by the Ladd band anomaly.^[4,13]^ In this case study, the patient mainly presented with vomiting episodes along with weight loss and right lower abdominal pain. These observations are consistent with literature reports. The relatively rare occurrence of the condition in adults and the atypical nature of the symptoms that vary greatly among patients pose significant challenges for diagnosis. Hence, intestinal malrotation is prone to misdiagnosis or missed diagnosis and is frequently mistaken for other abnormalities, such as peptic ulcer syndrome, irritable bowel syndrome, hepatobiliary pancreatic diseases, and psychiatric disorders.^[13,21]^

During the diagnostic process, it is imperative to distinguish this condition from the following ailments: Congenital duodenal atresia or stenosis: Patients present with persistent vomiting. Contrast imaging of the upper gastrointestinal tract reveals marked duodenal dilation without ectopic appendix or colon. However, B-ultrasonography or CT examination does not indicate any abnormalities in the mesenteric arteries and veins. Observation of the Treitz ligament position can differentiate incomplete obstruction caused by stenosis; Superior mesenteric artery syndrome: Vertical interruption of the barium column in the horizontal segment of the duodenum is evident on upper gastrointestinal contrast imaging, which forms a distinctive “pencil sign.” When the patient is positioned prone, barium passes smoothly, whereas retrograde peristalsis diminishes. Nevertheless, intestinal ectopia is not observed; Duodenal hernia is characterized by a localized abnormal aggregation of the small intestine, which forms a “belt-like” shape, thereby causing the compression and displacement of adjacent tissue structures. Moreover, mesenteric blood vessels protrude into the herniated intestinal segments, resulting in congestion toward the hernial opening; Annular pancreas: Clinical manifestations are very much similar to those of malrotation. Upper gastrointestinal contrast imaging shows narrowing in the descending segment of the duodenum without dilation. The Treitz’s ligament and the small intestine and colon are normally positioned. Contrast-enhanced CT scan demonstrates enhanced pancreatic tissue, which encircles the descending segment; Imaging examinations enable direct visualization of the extent of damage and local duodenal compression caused by malignant or benign tumors.^[10,20,22]^ However, these diagnoses should be ultimately confirmed via surgical intervention.^[10]^

The diagnosis of adult malrotation poses considerable challenges, and the incidence and mortality rates are higher than those in children. The diagnosis typically necessitates a series of imaging examinations and auxiliary diagnostic methods as physical examination and X-ray have limited diagnostic value.^[23]^ In this case, the patient was subjected to several investigations, including blood routine analysis, tumor marker assessment, upper abdominal CT scan, and complete gastrointestinal contrast study. The CT findings suggested small bowel torsion with intestinal obstruction. Furthermore, complete gastrointestinal contrast study revealed marked duodenal dilation, much further supported the possibility of small bowel obstruction. Relevant literature alludes that abdominal CT plays a pivotal role in preoperative diagnosis and is the preferred diagnostic modality owing to its enhanced accuracy.^[18,24]^ One distinctive radiological feature of small bowel volvulus that serves as a valuable diagnostic clue is the presence of a whirlpool-like change in the small intestine.^[25]^ Upper gastrointestinal contrast studies are standard imaging techniques to assess malrotation.^[18,24,26–29]^ However, as the findings are often similar to those of intestinal obstruction, additional laparoscopic confirmation is necessary. When adult patients have extensive medical histories involving complex adhesions and unclear anatomical levels, conventional open surgery or conversion to this surgical modality may be required.^[23]^ Overall, early and prompt surgical intervention upon symptom onset is necessary^[24]^ as delayed treatment can lead to severe complications, such as intestinal necrosis.^[30]^

Intestinal malrotation is characterized by the abnormal positioning of intestines, isolated mesenteric attachments, and incomplete rotation leading to intestinal obstruction. The exact cause for this problem is yet to be elucidated. However, pathologically, the problem manifests as an abnormally terminated embryonic intestinal rotation and mesenteric attachment, resulting in the compression of the duodenum and upper segment.^[18]^ Research on this condition is currently focused on children, and studies on adults are limited. Ladd procedure, named after Dr William Ladd who first proposed it in 1936, is the standard surgical treatment for this disease. The major steps include the following: complete release of membranous bands (Ladd bands) that adhere between the duodenum and cecum to eliminate intestinal obstruction; enlargement of the root of the mesentery to prevent further torsion of the small intestine; repositioning of the twisted small intestine in the opposite direction; assessment for necrotic bowel requiring resection; fixation of the duodenum along the left side of the abdomen, with the small intestine fixed on the right side and cecum/colon placed on the left side of the abdomen; simultaneous appendectomy to avoid misdiagnosis pertaining to alterations in cecal position.^[4,10,18,20,31–36]^ Postoperatively, fasting with fluid replacement is required until intestinal motility is restored before commencing oral intake.

Based on the conventional Ladd procedure, a combination of gastrojejunostomy and Braun anastomosis was implemented. Specifically, the distal jejunum dilated by the duodenum was selected, and a side-to-side anastomosis with the greater curvature of the stomach was performed to enhance the food outflow passage and prevent the delayed passage resulting from poor duodenal motility. Braun anastomosis effectively alleviates the efferent loop syndrome caused by gastrojejunostomy, diverts alkaline digestive fluids, assuages bile reflux, and reduces irritation to gastric mucosa. The success of this surgery can be determined within 6 months postoperatively based on symptom relief and weight gain in patients.^[26]^ According to a previous study, 89% of symptomatic patients with malrotation who underwent the procedure achieved complete resolution.^[11,33,37]^ In this case, substantial symptom relief was observed after surgical treatment, along with noticeable weight gain during the 6-month follow-up period.

## 3. Conclusion

Congenital intestinal malrotation is a rare condition in adults and presents significant diagnostic and therapeutic challenges owing to the lack of specific symptoms and clinical experience. Misdiagnosis, missed diagnosis, and delayed diagnosis are common occurrences, which lead to severe or even life-threatening consequences because of the secondary intestinal necrosis resulting from torsion. In this case report, a patient experiencing symptoms for 40 years and admitted with acute intestinal obstruction accompanied by intestinal torsion has been presented. Despite conservative gastric decompression and somatostatin administration, significant improvement was not observed in the patient’s condition. Surgical intervention ensured successful recovery without further gastrointestinal symptoms, such as nausea or vomiting. Considerable weight gain was also observed postoperatively. Based on the diagnostic and treatment process for this patient, it is necessary to consider congenital intestinal malrotation when dealing with bowel obstruction associated with torsion and perform appropriate examinations. Upper abdominal CT scan and complete gastrointestinal contrast studies can aid in the accurate diagnosis of this disease. Surgery remains the mainstay treatment for congenital intestinal malrotation, with Ladd procedure being the standard approach for managing such cases. Moderate modifications were made via conventional Ladd surgery, along with gastrojejunal and Braun anastomoses. Follow-up evaluations showed favorable therapeutic outcomes, confirming its safety and feasibility and justifying the wider adoption of the technique.

## Author contributions

Yin MD and Hao LL Contributed to manuscript writing and editing and picture collection; Li G, Li YT, and Xu BL contributed to the revision of the article; Chen XR contributed to conceptualization and supervision; all authors have read and approved the final manuscript.

Data curation: Meng-Di Yin, Li-Liang H.

Writing—original draft: Meng-Di Yin, Li-Liang Hao.

Writing—review and editing: Guang Li, Yu-Tao Li, Bao-Li Xu.

Conceptualization : Xin-Rui Chen.

Supervision: Xin-Rui Chen.
